# Markers of Biological Aging and Survival in Recurrent and Metastatic Head and Neck Cancer Patients Receiving Immunotherapy

**DOI:** 10.3390/epigenomes10040068

**Published:** 2026-10-19

**Authors:** Drew R. Nannini, Inamul Haque, Sushanta K. Banerjee, Suman Kambhampati

**Affiliations:** 1 Department of Hematology and Oncology, University of Kansas Medical Center, Kansas City, KS 66160, USA; 2 Department of Hematology and Oncology, Veterans Affairs Medical Center, Kansas City, MO 64128, USA; 3 Department of Cancer Biology, University of Kansas Medical Center, Kansas City, KS 66160, USA; 4 Cancer Research Unit, Veterans Affairs Medical Center, Kansas City, MO 64128, USA; 5 Department of Pathology, University of Kansas Medical Center, Kansas City, KS 66160, USA

**Keywords:** head and neck cancer, squamous cell carcinoma, DNA methylation, aging, epigenetics, immunotherapy, survival

## Abstract

Background/Objectives: Immunotherapy has become a cornerstone in the treatment of recurrent and metastatic head and neck squamous cell carcinoma (R/M HNSCC), but reliable noninvasive biomarkers of prognosis remain limited. We investigated the associations between markers of biological aging and survival outcomes among patients with R/M HNSCC treated with immune checkpoint inhibitors (ICIs). Methods: Peripheral blood samples collected prior to ICI initiation from 100 patients with R/M HNSCC underwent DNA methylation profiling using the Illumina Infinium MethylationEPIC array. Four epigenetic age acceleration measures were estimated from DNA methylation levels and evaluated for associations with progression-free survival (PFS) and overall survival (OS). Results: Greater intrinsic and extrinsic epigenetic age acceleration were each associated with longer PFS (*p* = 0.025 and *p* = 0.048, respectively) and OS (*p* = 0.024 and *p* = 0.029, respectively). Significant interactions between GrimAge acceleration and chronological age were observed for both PFS (*p* = 0.010) and OS (*p* < 0.001). Greater GrimAge acceleration was associated with improved PFS among patients less than 65 years of age but shorter OS among patients 65 years of age or older. Conclusions: In this exploratory study, pretreatment epigenetic aging measures were associated with survival outcomes among patients with R/M HNSCC receiving ICIs. These findings suggest biological aging metrics may have clinical relevance and warrant further investigation as potential noninvasive prognostic biomarkers.

## 1. Introduction

The incidence of head and neck squamous cell carcinoma (HNSCC) has increased in the United States over the past decade, with approximately 60,000 new cases projected in 2026 [1], reflecting a persistent and growing public health burden. Although the majority of patients initially present with localized disease, nearly 14% have metastatic disease at the time of diagnosis and an additional 20–30% will develop metastatic disease following curative-intent therapy [2]. Historically, treatment options for recurrent and metastatic (R/M) HNSCC not amenable to local therapy were limited to cetuximab in combination with platinum-based chemotherapy, yielding a median overall survival (OS) of 10 months [3]. Survival among these patients further improved with the introduction of immune checkpoint inhibitors (ICIs) [4,5]. Despite the addition of ICIs, survival outcomes remain limited, with a median OS of 13 months [6]. Given the modest improvement in survival with current therapies, identifying patients most likely to benefit from ICIs remains an unmet need in the management of R/M HNSCC.

As cancer care increasingly shifts toward precision medicine, prognostic biomarkers have emerged as important tools to guide therapeutic decision-making. Currently, the U.S. Food and Drug Administration endorses several biomarkers for predicting response to ICIs, including programmed death-ligand 1 (PD-L1), microsatellite instability/deficient mismatch repair, and tumor mutational burden [7]. While higher PD-L1 expression has been associated with favorable outcomes in several cancers, its predictive performance remains limited [8], and its expression has shown inconsistent associations with clinical benefit among patients with HNSCC [9]. Moreover, these biomarkers require invasive tissue biopsies, which increase procedural risk, patient discomfort, and healthcare costs. These limitations have generated growing interest in noninvasive, blood-based biomarkers capable of improving prognostication and potentially guiding treatment selection. Among these emerging biomarkers, DNA methylation based-biomarkers have shown promise for cancer prognosis and treatment response prediction.

One such DNA methylation–based biomarker is biological age, which reflects an individual’s physiological state and provides information beyond chronological age alone [10]. Epigenetic age acceleration, defined as the discrepancy between chronological and biological age, has been associated with progression-free survival (PFS) and OS in several cancers, including HNSCC patients treated with radiation therapy [11,12,13]. Additionally, DNA methylation-derived immune cell profiles have been associated with survival outcomes among patients with R/M HNSCC receiving ICIs [14]. In contrast to these profiles, which characterize circulating immune cell composition, epigenetic aging metrics capture distinct aspects of age-related biological processes, including immunosenescence, physiological function, and comorbidities [15]. These metrics may provide complementary prognostic information by capturing host-level factors that may influence disease progression and treatment response beyond those reflected by immune cell composition. Therefore, given the need for improved prognostic tools in immunotherapy-treated HNSCC and the potential relevance of aging-related epigenetic signatures, the purpose of this exploratory study was to investigate the association between markers of biological age and survival outcomes among patients with R/M HNSCC treated with ICIs.

## 2. Results

### 2.1. Patient Characteristics

Table 1 presents the descriptive characteristics for the 100 patients included in this analysis. Overall, the average chronological age was 64.4 years and 75% of patients were male. The average PD-L1 CPS was 40.8 and the median PFS and OS for patients were 132 and 538 days, respectively.

### 2.2. Biological Age and Survival Outcomes

**Figure 1:**
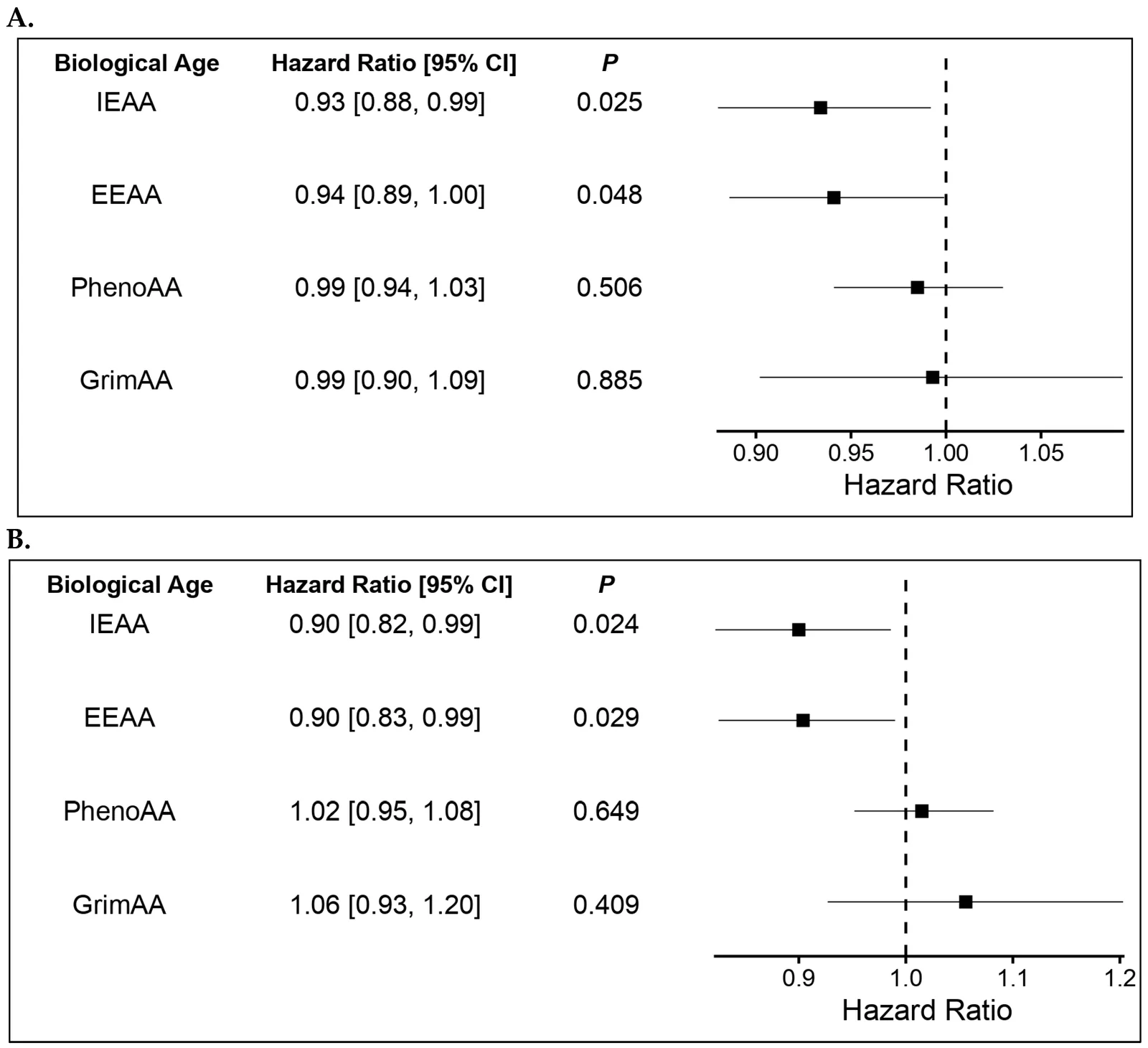
Hazard ratios for the associations between the markers of biological aging and progression-free survival (**A**) and overall survival (**B**) in R/M HNSCC.

Figure 1 presents the results for the association between the markers of biological age and survival outcomes. IEAA and EEAA were significantly and inversely associated with PFS (Figure 1A). Specifically, each one-year increase in IEAA and EEAA was associated with a 7% (HR 0.93; 95% CI: 0.88, 0.99; *p* = 0.025) and 6% (HR 0.94; 95% CI: 0.89, 1.00; *p* = 0.048) lower hazard of disease progression, respectively. IEAA and EEAA were also significantly and inversely associated with OS (Figure 1B). Each one-year increase in IEAA and EEAA was associated with a 10% (HR 0.90; 95% CI: 0.82, 0.99; *p* = 0.024) and 10% (HR 0.90; 95% CI: 0.83, 0.99; *p* = 0.029) lower hazard of death, respectively. PhenoAA and GrimAA were not associated with either PFS or OS.

### 2.3. Biological Age and Survival Outcomes by Chronological Age

**Figure 2:**
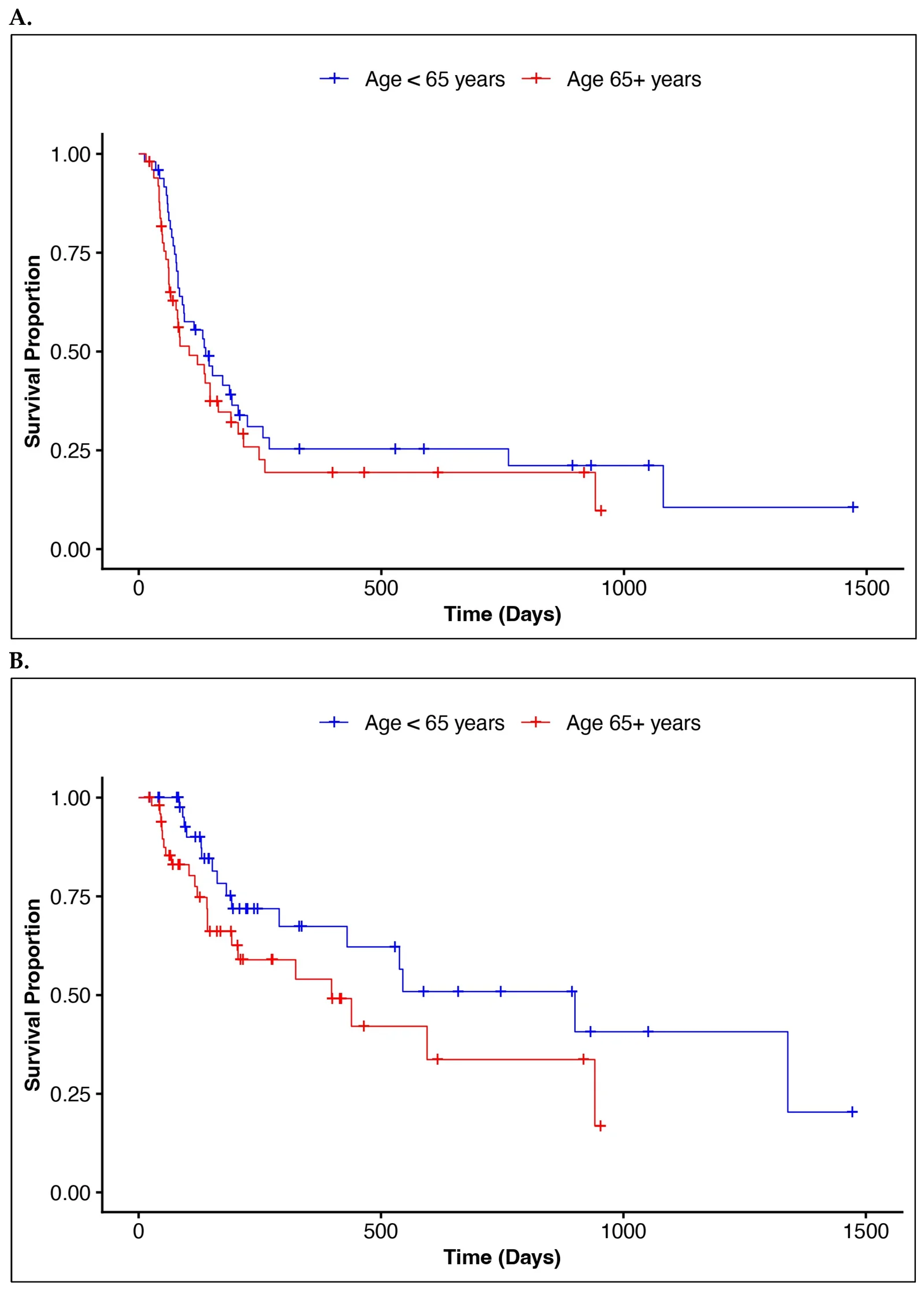
Kaplan–Meier curves for progression-free survival (**A**) and overall survival (**B**) in R/M HNSCC by chronological age.

**Figure 3:**
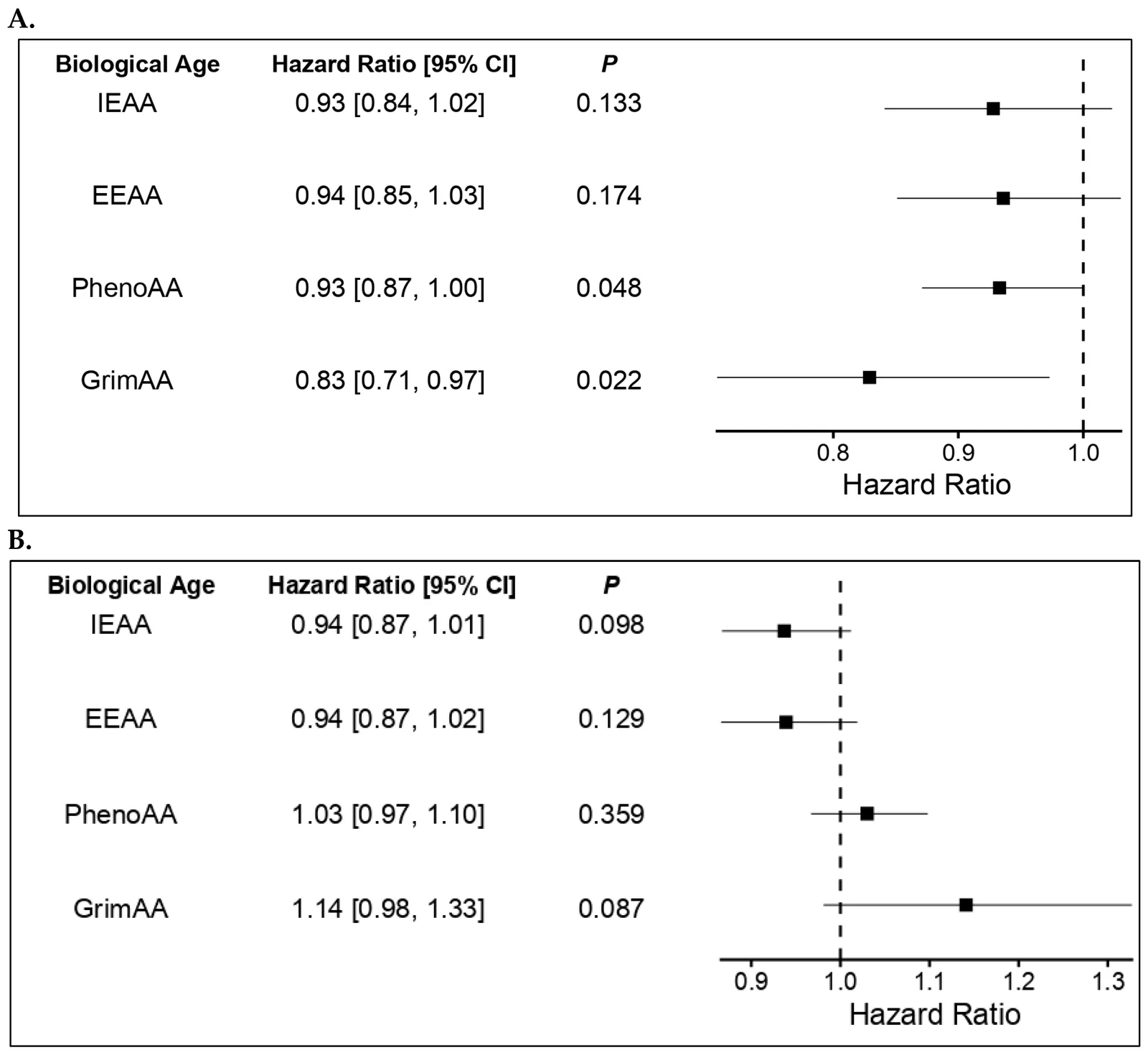
Hazard ratios for the associations between the markers of biological aging and progression-free survival in R/M HNSCC among patients (**A**) less than 65 years of age and (**B**) 65 years of age or older.

**Figure 4:**
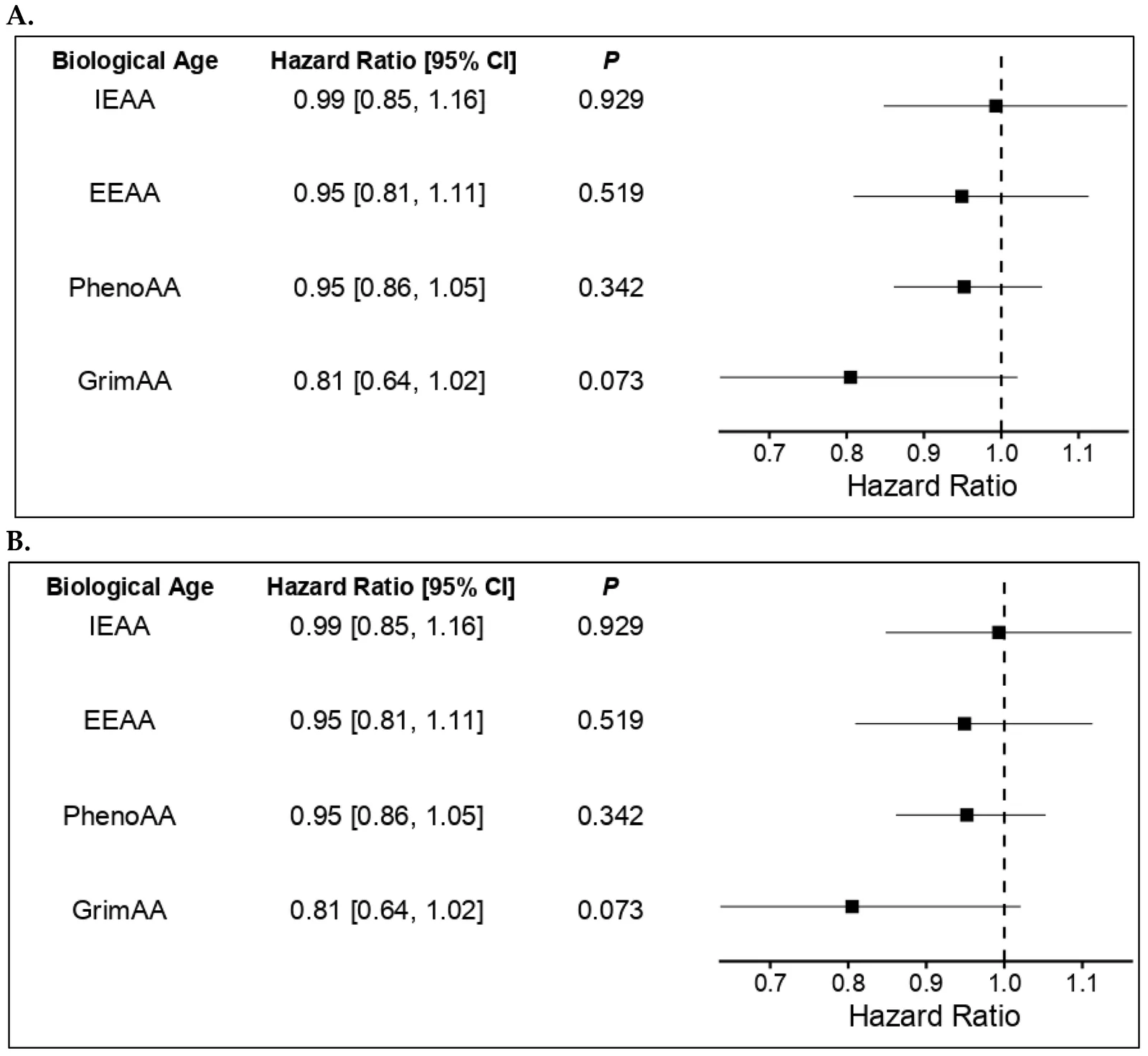
Hazard ratios for the associations between the markers of biological aging and overall survival in R/M HNSCC among patients (**A**) less than 65 years of age and (**B**) 65 years of age or older.

Figure 2 presents the Kaplan–Meier curves for PFS (Figure 2A) and OS (Figure 2B) by median chronological age. No significant differences in survival were observed between the strata (log-rank *p* = 0.277 and *p* = 0.086, respectively). During Cox proportional hazards modeling, significant statistical interactions between chronological age and PhenoAA (*p* = 0.041) and GrimAA (*p* = 0.010) with PFS and GrimAA (*p* < 0.001) with OS were observed (Supplemental Table S1). Figure 3 presents the stratified results by chronological age for the associations between the markers of biological age and PFS. Among patients less than 65 years of age (Figure 3A), each one-year increase in PhenoAA and GrimAA was associated with a 7% (HR 0.93; 95% CI: 0.87, 1.00; *p* = 0.048) and 17% (HR 0.83; 95% CI: 0.71, 0.97; *p* = 0.022) lower hazard of disease progression, respectively. No significant associations with PFS were observed among patients 65 years of age or older (Figure 3B). Figure 4 presents the stratified results by chronological age for the associations between the markers of biological age and OS. Among patients 65 years of age or older (Figure 4B), each one-year increase in GrimAA was associated with a 27% (HR 1.27; 95% CI: 1.03, 1.58; *p* = 0.026) greater hazard of death. No significant associations were observed among patients less than 65 years of age with OS (Figure 4A). No significant differences were observed in PFS and OS when patients were stratified by either sex (Supplemental Figure S1, log-rank *p* = 0.332 and *p* = 0.242, respectively) or median PD-L1 CPS (Supplemental Figure S2, log-rank *p* = 0.681 and *p* = 0.831, respectively) as assessed by Kaplan–Meier curves. No significant interactions were observed between either sex (Supplemental Table S2 and Supplemental Figures S3 and S4) or median PD-L1 CPS (Supplemental Table S3 and Supplemental Figures S5 and S6) and the markers of biological age with PFS or OS during Cox proportional hazards modeling.

### 2.4. Surrogate Biomarkers of Plasma Proteins of GrimAge and Survival Outcomes

The eight DNA methylation surrogate biomarkers of plasma proteins of GrimAge were assessed to better examine the associations between GrimAA and the survival outcomes observed during stratified analyses by chronological age. Beta-2 microglobulin (B2M; *p* = 0.018) and tissue inhibitor metalloproteinase 1 (TIMP1; *p* = 0.017) were significantly associated with PFS among patients less than 65 years of age, while no surrogate biomarkers were associated among patients 65 years of age or older (Supplemental Figure S7). Additionally, leptin (*p* = 0.045) was significantly associated with OS among patients less than 65 years of age and B2M (*p* = 0.048) and plasminogen activation inhibitor 1 (PAI-1; *p* = 0.001) were significantly associated with OS in patients 65 years of age or older (Supplemental Figure S8).

## 3. Discussion

In this exploratory study, we observed that markers of biological aging were significantly associated with PFS and OS among patients with R/M HNSCC receiving ICIs. Greater IEAA and EEAA were associated with longer PFS and OS, whereas the association between GrimAA and survival differed by chronological age. Our findings add to the current literature by suggesting that epigenetic aging metrics may be associated with survival outcomes among patients with R/M HNSCC and support further investigation of pretreatment epigenetic aging markers as potential noninvasive prognostic biomarkers in this population.

Aging is characterized as a progressive, multifactorial decline in physiological function and has been associated with worse survival outcomes [16]. Although chronological age has traditionally served as the standard metric for evaluating health and aging, growing evidence has suggested biological age may be a superior prognostic marker of health outcomes, morbidity, and mortality [17,18]. In the current analysis, greater IEAA and EEAA were associated with longer PFS and OS. IEAA reflects intrinsic properties of the aging process across cell types and organs, independent of age-related changes in cell composition and environmental factors [15]. While the mechanisms underlying these findings remain unclear, one potential explanation may involve age-related alterations in mitochondrial function. Emerging evidence suggests that biological age is associated with mitochondrial dysfunction, which can activate downstream innate immune signaling pathways such as cGAS-STING [19,20]. Activation of this pathway has been implicated in antitumor immunity and may enhance responses to PD-1 blockade [21]. EEAA, in contrast, reflects age-related immune remodeling, including changes in leukocyte composition characteristic of immunosenescence, e.g., expansion of exhausted T cell populations [22]. A hallmark of exhausted T cells is expression of numerous inhibitory receptors, most notably PD-1, and blockade of the PD-1/PD-L1 inhibitory pathway can restore T cells’ ability to proliferate, secrete cytokines, and exert cytotoxic effects [23,24]. In the current study, patients with higher EEAA may have improved PFS and OS due to age-related expansion of T-cell populations expressing PD-1 and other inhibitory receptors, potentially creating an immune landscape more responsive to PD-1/PD-L1 blockade. Together, our findings suggest cellular intrinsic and immunological aging metrics are associated with PFS and OS and may have implications as prognostic biomarkers for survival among R/M HNSCC patients.

Unlike IEAA and EEAA, GrimAA incorporates surrogate plasma proteins associated with mortality and morbidity, potentially capturing a broader range of biological processes. We observed significant interactions between GrimAA and chronological age for both PFS and OS, with greater GrimAA associated with improved survival among patients less than 65 years of age but worse survival among those aged 65 years or older. These age-dependent associations may reflect differences in comorbidity burden, physiologic reserve, or other age-related health factors captured by the plasma protein surrogates. For example, B2M, TIMP-1, leptin, and PAI-1 were significantly associated with survival outcomes during stratified analyses. B2M is an integral component of major histocompatibility complex class I molecules that are required for the presentation of intracellular antigens to cytotoxic T cells and loss of B2M impairs tumor antigen presentation and is a well-established mechanism of resistance to immune checkpoint inhibition [25]. The association between GrimAA and PFS observed in the current study may be partially explained by pathways related to preserved tumor–immune recognition and enhanced susceptibility to PD-1/PD-L1 blockade via B2M. TIMP-1 is an inhibitory molecule that plays a role in extracellular matrix composition and has been associated with poorer survival among HNSCC patients [26]. Recent evidence, however, has demonstrated that TIMP-1 is associated with immune checkpoint molecules and immunotherapy efficacy [27], suggesting TIMP-1 may reflect biological processes relevant to immune checkpoint activity and treatment response in HNSCC. Leptin is a peptide hormone involved in appetite regulation, energy homeostasis, and obesity. Although obesity promotes chronic inflammation, tumor progression, and PD-1-mediated T-cell dysfunction, leptin-induced PD-1 upregulation may increase tumor dependence on the PD-1 immune checkpoint pathway, potentially contributing to improved efficacy and survival outcomes observed with PD-1/PD-L1 blockade in patients [28]. Lastly, PAI-1 is a multifunctional regulator of fibrinolysis that has been implicated in tumor progression, angiogenesis, and immune suppression [29]. While elevated PAI-1 is generally associated with an immunosuppressive tumor microenvironment, its positive correlation with PD-L1 expression raises the possibility that tumors with higher PAI-1 levels may exhibit greater reliance on PD-1/PD-L1-mediated immune escape, potentially influencing responsiveness to immune checkpoint inhibition [30]. In sum, these GrimAge surrogate plasma proteins may explain the association in survival outcomes among R/M HNSCC patients receiving ICI.

While greater biological aging has generally been associated with worse survival outcomes [31,32], favorable associations have also been reported. For example, favorable associations between DNA methylation aging measures and survival have been reported in patients with cervical squamous cell carcinoma and gliomas [12,33]. In our study, the observed hazard ratios corresponded to approximately 10–20% differences in the hazard of progression or death, depending on the biological aging metric and patient age. Although these findings suggest potential prognostic relevance, the clinical significance remains uncertain, and the prognostic value of these biological aging metrics beyond clinical factors has not been established. If validated, pretreatment blood-based epigenetic aging measures could potentially complement established clinical and tumor-related prognostic factors to improve risk stratification among patients with R/M HNSCC receiving ICIs. Overall, these findings suggest the associations between biological aging and survival may be cancer- and context-specific, underscoring the need for validation in independent cohorts before these measures can be considered for clinical implementation.

Although this study identified novel associations between age-related changes to the epigenome and survival among R/M HNSCC patients treated with ICIs, this study is not without limitations. The sample size of the current study is small, reducing the power to identify additional associations between the markers of biological aging and survival. Additional covariates, such as human papillomavirus status, metastatic disease burden, prior treatment history, and comorbidities, were unavailable and may have introduced residual confounding that could influence the direction and magnitude of the observed associations. Therefore, caution is warranted when interpreting these findings. Furthermore, the type of ICI, e.g., pembrolizumab and nivolumab, was not available, limiting the ability to determine whether the associations differed based on the type of immunotherapy administered. Due to the number of independent and dependent variables examined, this study exhibited multiple analyses, which may increase the risk of false positive associations. Correction for multiple testing was not performed as this study was exploratory and to avoid hindering novel associations in this hypothesis-generating study [34]. However, caution should be exercised to avoid over-interpreting these findings. Lastly, independent replication was not performed as no comparable publicly available dataset containing pretreatment blood DNA methylation profiles and corresponding survival outcomes for patients with R/M HNSCC treated with ICIs was identified. Replication of these findings in larger, prospective cohorts with further adjustment for relevant clinical and prognostic covariates is warranted to confirm the observed associations.

## 4. Materials and Methods

### 4.1. Patient Study Population

The following analyses were conducted using a publicly available dataset, with details on patient identification, clinical follow-up, and peripheral blood processing previously described [14]. Briefly, patients with R/M HNSCC were recruited for a prospective, multi-center study at Dana-Farber Cancer Institute, Dartmouth Cancer Center, and Rhode Island Hospital. Eligible patients were at least 18 years of age with a plan to initiate ICI therapy as determined by the treating medical oncologist with no restrictions on prior therapy. Patients were excluded if they had carcinoma in situ, cutaneous malignancies, or salivary gland cancers.

### 4.2. Blood DNA Methylation Profiling

Peripheral blood samples were collected prior to ICI initiation and transported to the Laboratory for Molecular Medicine at Brown University for DNA extraction and processing. DNA methylation profiling was performed using the Illumina Infinium MethylationEPIC v1 and v2 array platforms and raw IDAT files were obtained for analysis. The R package *minfi* (v1.54.1) was used for data preprocessing and quality control [35]. Samples and probes with detection *p*-values > 0.01 were excluded. Background correction and dye-bias normalization were performed using the Noob method. The arrays were merged by retaining overlapping probes and batch effects were corrected using ComBat [36].

### 4.3. Estimation of Markers of Biological Aging

Four principal component-based aging metrics were estimated and analyzed via the R scripts *PC Clocks* using DNA methylation levels: intrinsic epigenetic age acceleration (IEAA), extrinsic epigenetic age acceleration (EEAA), PhenoAge acceleration (PhenoAA), and GrimAge acceleration (GrimAA) [37]. IEAA was estimated using 353 CpG probes and has previously been associated with cell-intrinsic aging [38]. EEAA was calculated using 71 CpG probes and has been associated with immunological aging [39]. PhenoAA was estimated using 513 CpG probes and has previously been associated with age-related health conditions [40]. GrimAA was estimated from 1030 CpG probes and has been associated with lifespan [41]. The use of principal component-based clocks minimizes technical variability by leveraging stable methylation patterns rather than individual CpG sites, thereby improving reproducibility and cross-platform robustness.

### 4.4. Statistical Analysis

Cox proportional hazards models were used to evaluate the associations between the markers of biological aging (independent variables) and PFS and OS (dependent variables). Chronological age, sex, and a blood methylation-based smoking score [42] were included as covariates to control for confounding given their well-established influence on DNA methylation profiles and clinical outcomes [14]. Interaction and stratified analyses were performed by chronological age, sex, and PD-L1 CPS. Kaplan–Meier curves were generated and log-rank testing was performed to evaluate for differences between strata. Cox proportional hazards models were further used to assess the relationship between GrimAge DNA methylation-based surrogate biomarkers of plasma proteins with PFS and OS. The proportional hazards assumption was assessed for each model, and appropriate adjustments were made when violations were identified. Associations were considered significant if *p* ≤ 0.05. All statistical analyses were performed using SAS Studio (v9.4; SAS Institute, Inc., Cary, NC, USA) and figures were generated using R version 4.5.1.

## 5. Conclusions

In summary, we identified associations between markers of biological aging and PFS and OS, as well as significant interactions with chronological age, among patients with R/M HNSCC receiving ICIs in this exploratory study. These findings provide preliminary evidence that pretreatment epigenetic aging measures may be associated with survival outcomes in this patient population and may serve as noninvasive prognostic biomarkers. However, given the limited sample size, potential residual confounding, and absence of a validation dataset, these findings should be considered hypothesis-generating. Validation in larger, prospective cohorts with additional clinical and prognostic data is warranted to demonstrate reproducibility and potential clinical relevance of these biomarkers before incorporation into clinical decision-making.

## Figures and Tables

**Table 1:** Patient demographic and clinical characteristics.

Patient Characteristics	N = 100
Chronological Age, mean (SD), years	64.4 (11.8)
Sex, n (%)	
Female	25 (25.0)
Male	75 (75.0)
Recruitment Center, n (%)	
Dana-Farber Cancer Institute	88 (88.0)
Dartmouth Cancer Center	10 (10.0)
Rhode Island Hospital	2 (2.0)
Location of Primary Tumor, n (%)	
Oral cavity	44 (44.0)
Oropharynx	34 (34.0)
Hypopharynx	8 (8.0)
Larynx	7 (7.0)
Paranasal sinuses and nasal cavity	3 (3.0)
Unknown	4 (4.0)
Smoking History, n (%)	
Current/Former	51 (51.0)
Never	40 (40.0)
Missing	9 (9.0)
ICI Treatment, n (%)	
Immunotherapy Only	79 (79.0)
Immunotherapy + Chemotherapy	20 (20.0)
Immunotherapy + EGFRi	1 (1.0)
Vital Status, n (%)	
Alive	63 (63.0)
Deceased	37 (37.0)
Progression-Free Survival, median, days	132
Overall Survival, median, days	538
PD-L1 CPS, mean (SD), score *	40.8 (35.0)
IEAA, mean (SD), years	0.00 (4.5)
EEAA, mean (SD), years	0.00 (4.2)
PhenoAA, mean (SD), years	0.00 (5.3)
GrimAA, mean (SD), years	0.00 (3.4)

CPS, combined positive score; IEAA, intrinsic epigenetic age acceleration; EEAA, extrinsic epigenetic age acceleration; PhenoAA, PhenoAge acceleration; GrimAA, GrimAge acceleration. * 91 patients had available PD-L1 CPS.

## Data Availability

The dataset used in this analysis is available in the Gene Expression Omnibus database, https://www.ncbi.nlm.nih.gov/geo/query/acc.cgi?acc=GSE277573 (accessed on 1 July 2025).

## Supplementary Materials

The following supporting information can be downloaded at: https://www.mdpi.com/article/10.3390/epigenomes10040068/s1, Figure S1: Kaplan-Meier curves for (A) progression free survival and (B) overall survival by sex; Figure S2: Kaplan-Meier curves for (A) progression free survival and (B) overall survival by PD-L1 CPS stratified at 30; Figure S3: Hazard ratios for the associations between the markers of biological aging and progression free survival in R/M HNSCC stratified by sex (A) females and (B) males; Figure S4: Hazard ratios for the associations between the markers of biological aging and overall survival in R/M HNSCC stratified by sex (A) females and (B) males; Figure S5: Hazard ratios for the associations between the markers of biological aging and progression free survival in R/M HNSCC stratified by PD-L1 CPS (A) less than 30 and (B) greater or equal to 30; Figure S6: Hazard ratios for the associations between the markers of biological aging and overall survival in R/M HNSCC stratified by PD-L1 CPS (A) less than 30 and (B) greater or equal to 30; Figure S7: Hazard ratios for the associations between the blood-based plasma proteins of GrimAge and progression free survival in R/M HNSCC among patients (A) less than 65 years of age and (B) 65 years of age or older; Figure S8: Hazard ratios for the associations between the blood-based plasma proteins of GrimAge and overall survival in R/M HNSCC among patients (A) less than 65 years of age and (B) 65 years of age or older; Table S1: Product terms for the associations between the markers of biological aging and median chronological age on survival outcomes in R/M HNSCC; Table S2: Product terms for the associations between the markers of biological aging and sex on survival outcomes in R/M HNSCC; Table S3: Product terms for the associations between the markers of biological aging and median PD-L1 CPS on survival outcomes in R/M HNSCC.
