# Supplementary material for: Markers of Biological Aging and Survival in Recurrent and Metastatic Head and Neck Cancer Patients Receiving Immunotherapy
Source: Epigenomes. 2026 Oct 19;10(4):68. doi: 10.3390/epigenomes10040068 (PMC13692446; doi:10.3390/epigenomes10040068)
Supplement: Supplementary file 1 [file epigenomes-10-00068-s001.zip › epigenomes-4590840-supplementary.pdf]

Supplemental Table S1. Product terms for the associations between the markers of biological aging and median chronological age on survival outcomes in R/M HNSCC

	P_{PFS}	P_{OS}
IEAA	0.866	0.122
EEAA	0.916	0.396
PhenoAA	0.041	0.193
GrimAA	0.010	<0.001

Chronological age was stratified at the median of 65 years of age

Supplemental Table S2. Product terms for the associations between the markers of biological aging and sex on survival outcomes in R/M HNSCC

	P_{PFS}	P_{OS}
IEAA	0.901	0.290
EEAA	0.578	0.566
PhenoAA	0.374	0.294
GrimAA	0.991	0.176

Supplemental Table S3. Product terms for the associations between the markers of biological aging and median PD-L1 CPS on survival outcomes in R/M HNSCC

	P_{PFS}	P_{OS}
IEAA	0.293	0.753
EEAA	0.459	0.985
PhenoAA	0.139	0.058
GrimAA	0.371	0.731

PD-L1 CPS was stratified at the median of 30

Supplemental Figure S1. Kaplan-Meier curves for (**A**) progression free survival and (**B**) overall survival by sex

A.

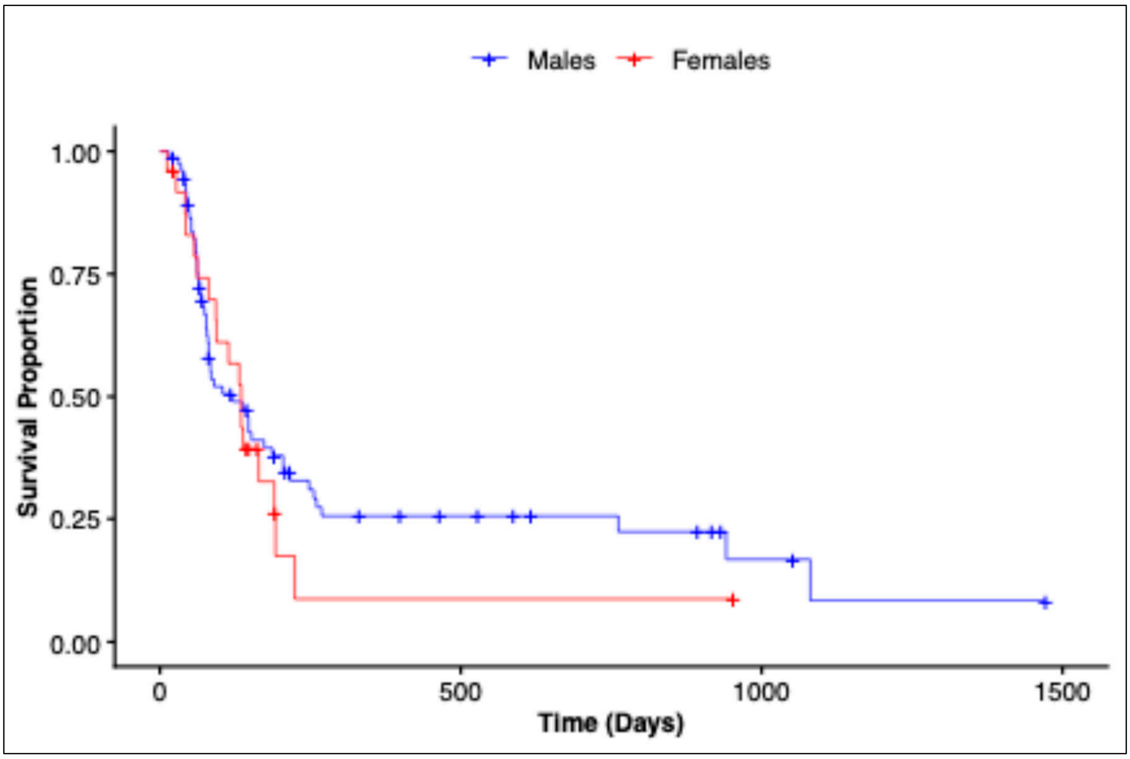

B.

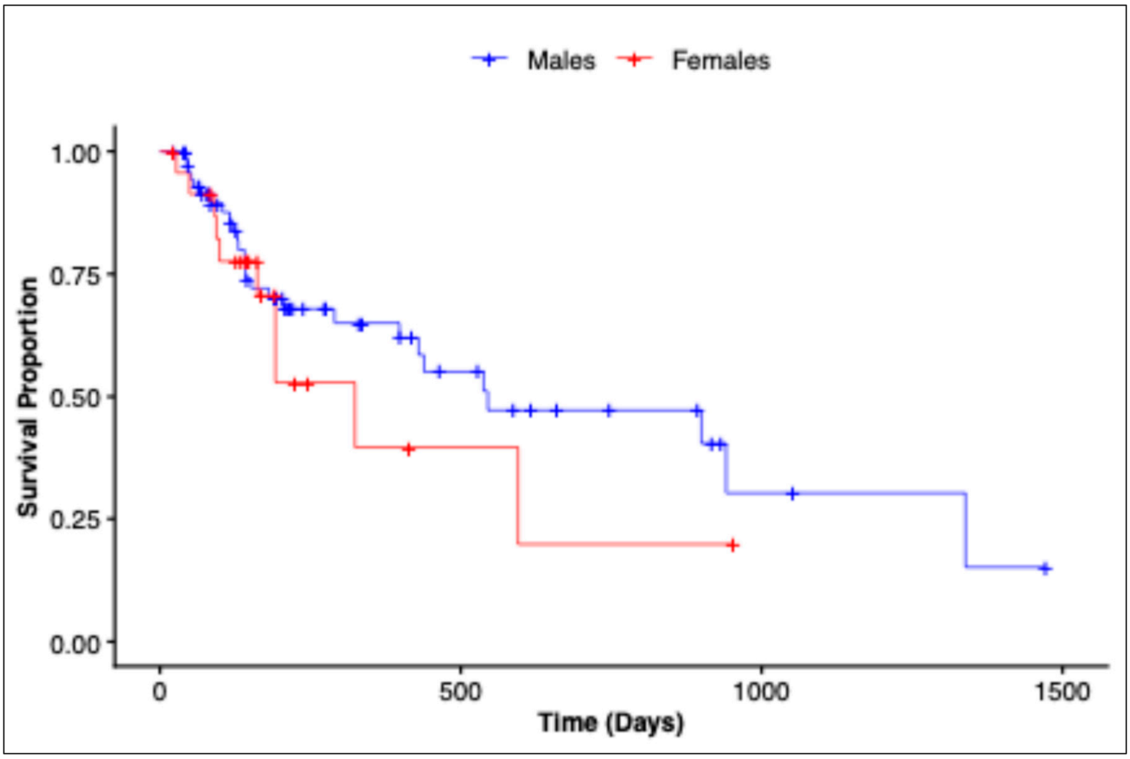

Supplemental Figure S2. Kaplan-Meier curves for (**A**) progression free survival and (**B**) overall survival by PD-L1 CPS stratified at 30

A.

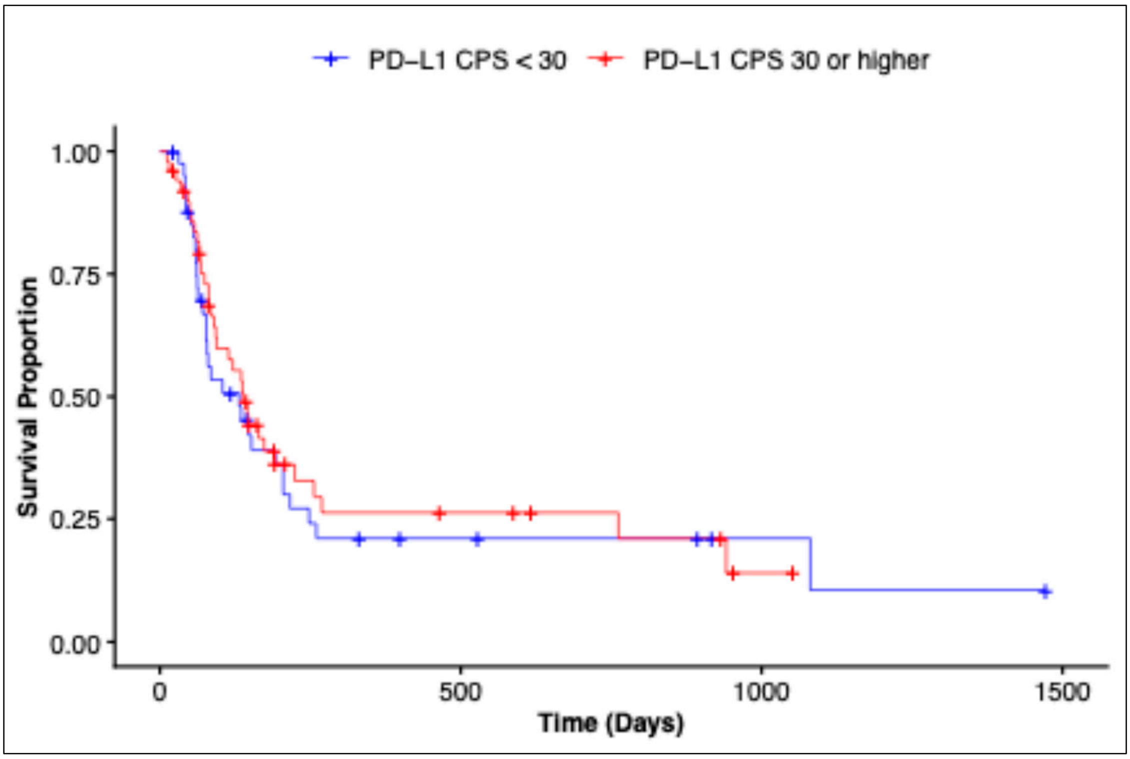

B.

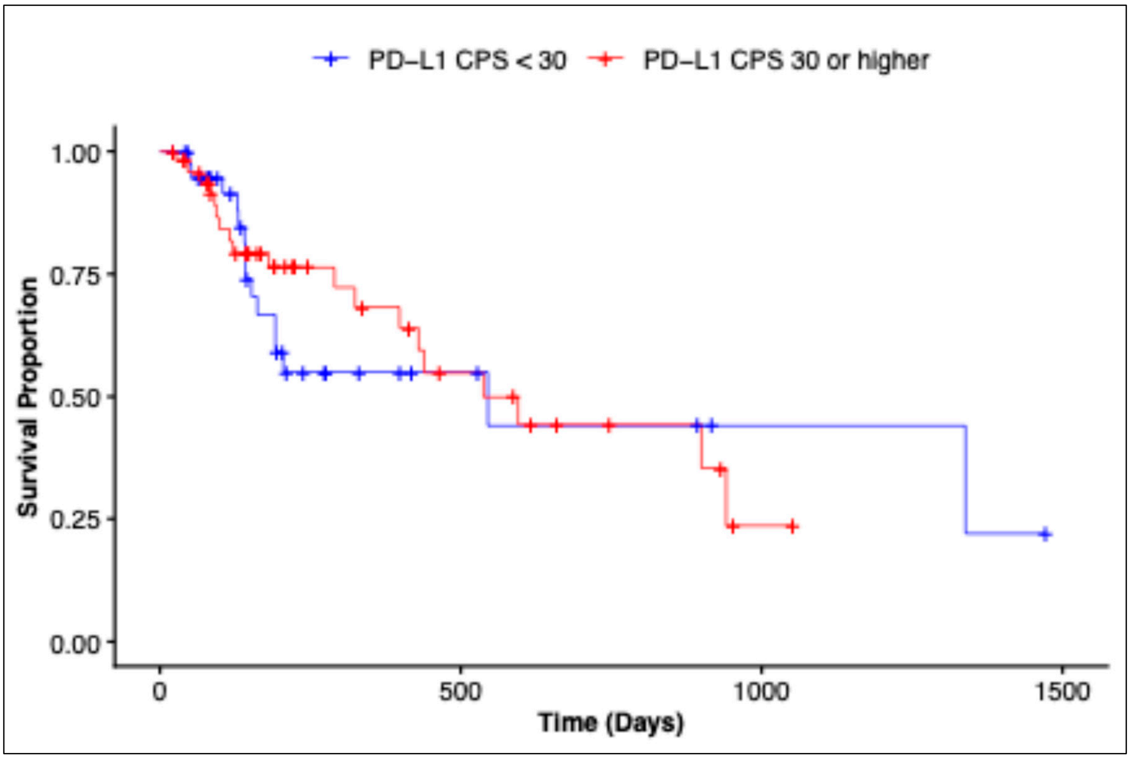

Supplemental Figure S3. Hazard ratios for the associations between the markers of biological aging and progression free survival in R/M HNSCC stratified by sex (**A**) females and (**B**) males

A.

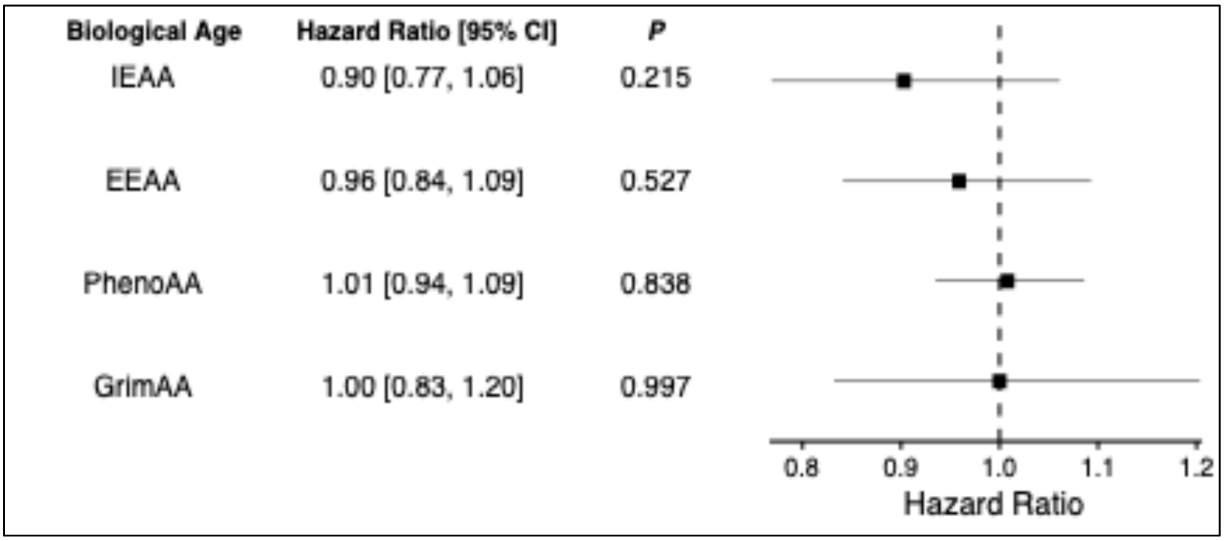

B.

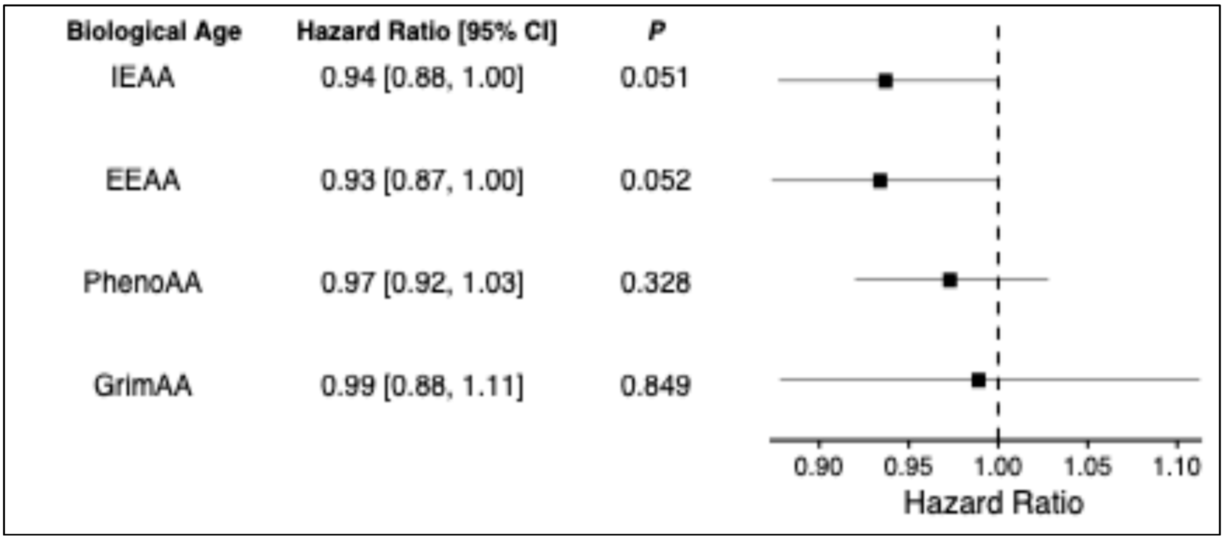

Supplemental Figure S4. Hazard ratios for the associations between the markers of biological aging and overall survival in R/M HNSCC stratified by sex (**A**) females and (**B**) males

A.

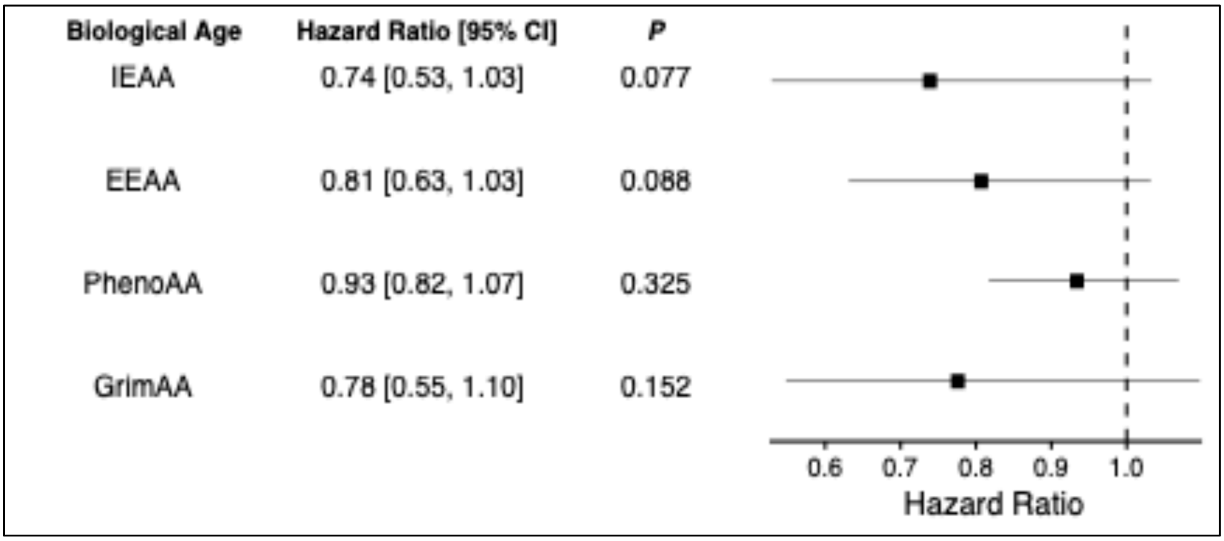

B.

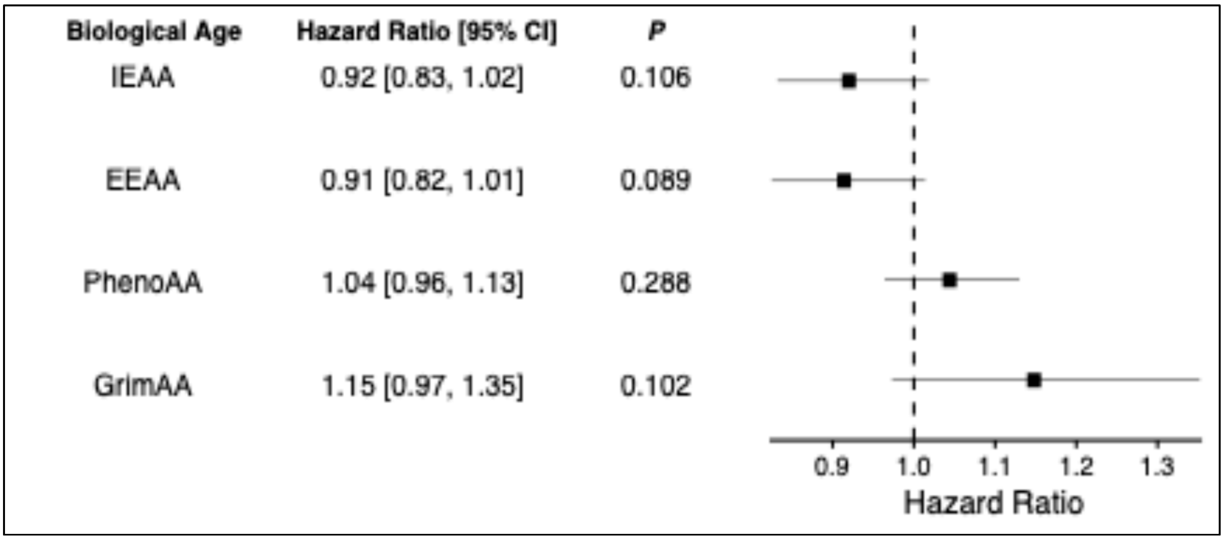

Supplemental Figure S5. Hazard ratios for the associations between the markers of biological aging and progression free survival in R/M HNSCC stratified by PD-L1 CPS (**A**) less than 30 and (**B**) greater or equal to 30

A.

Biological Age	Hazard Ratio [95% CI]	*P*
IEAA	0.92 [0.83, 1.01]	0.064
EEAA	0.93 [0.85, 1.02]	0.129
PhenoAA	0.95 [0.87, 1.03]	0.201
GrimAA	1.04 [0.88, 1.23]	0.643

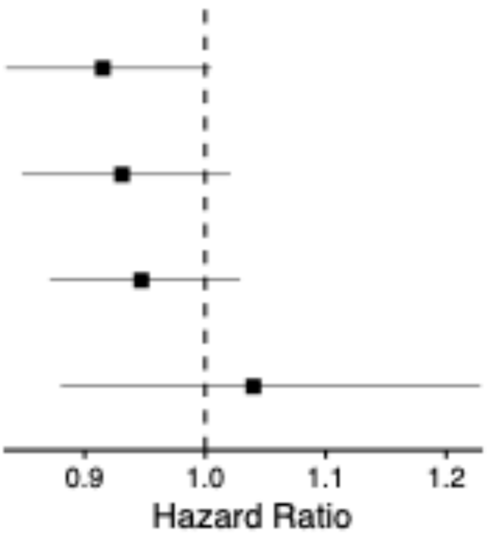

B.

Biological Age	Hazard Ratio [95% CI]	*P*
IEAA	0.99 [0.89, 1.11]	0.907
EEAA	0.99 [0.89, 1.09]	0.784
PhenoAA	1.02 [0.96, 1.09]	0.513
GrimAA	0.99 [0.86, 1.13]	0.844

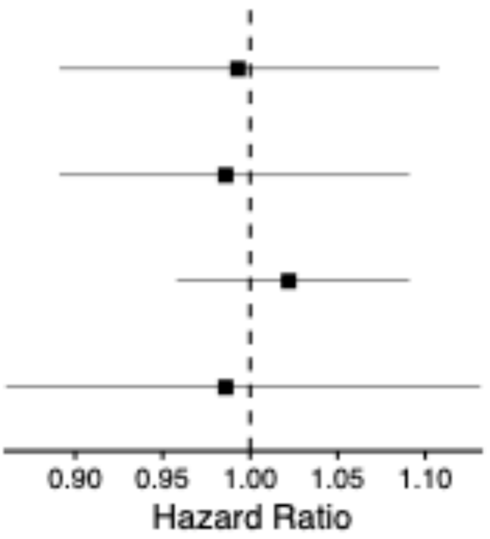

Supplemental Figure S6. Hazard ratios for the associations between the markers of biological aging and overall survival in R/M HNSCC stratified by PD-L1 CPS (**A**) less than 30 and (**B**) greater or equal to 30

A.

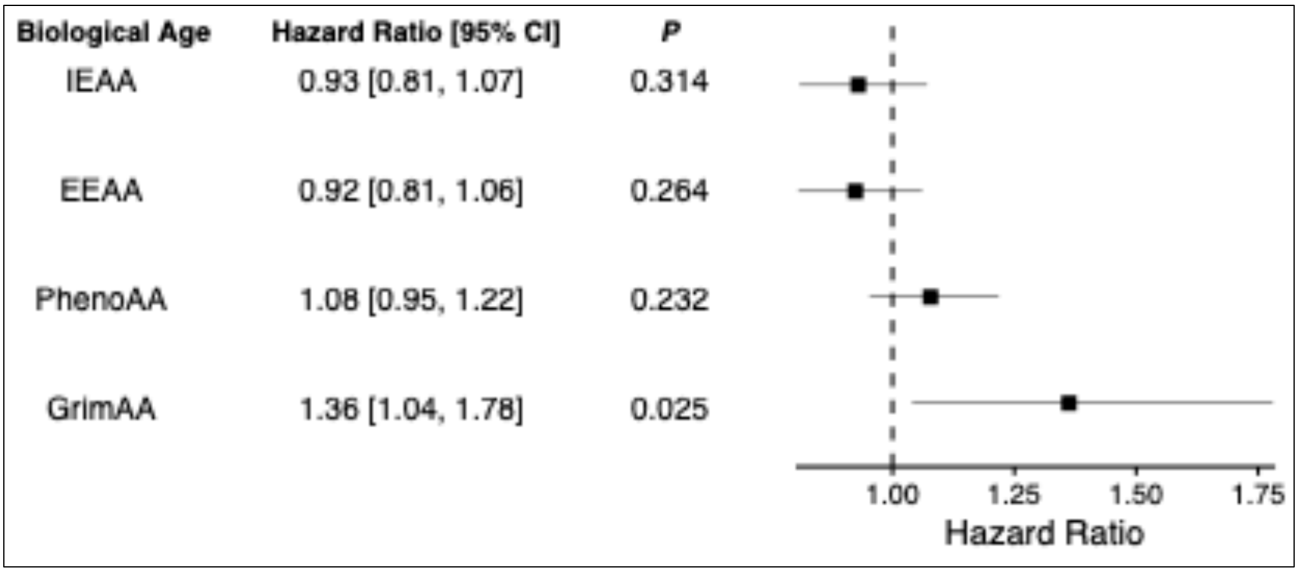

B.

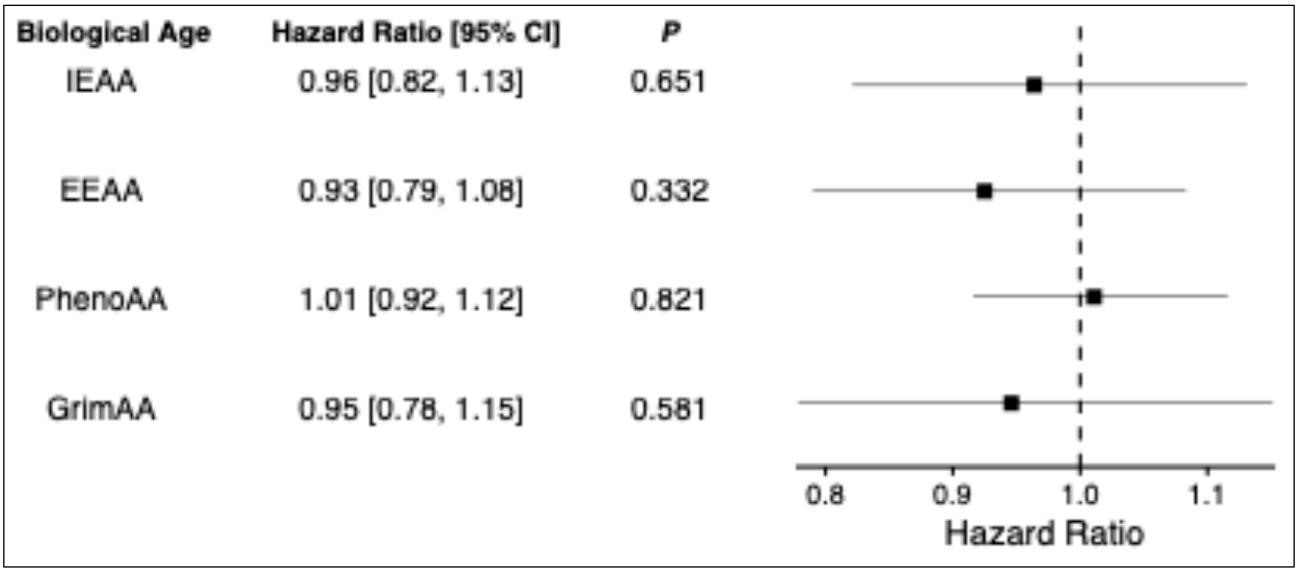

Supplemental Figure S7. Hazard ratios for the associations between the blood-based plasma proteins of GrimAge and progression free survival in R/M HNSCC among patients (**A**) less than 65 years of age and (**B**) 65 years of age or older

A.

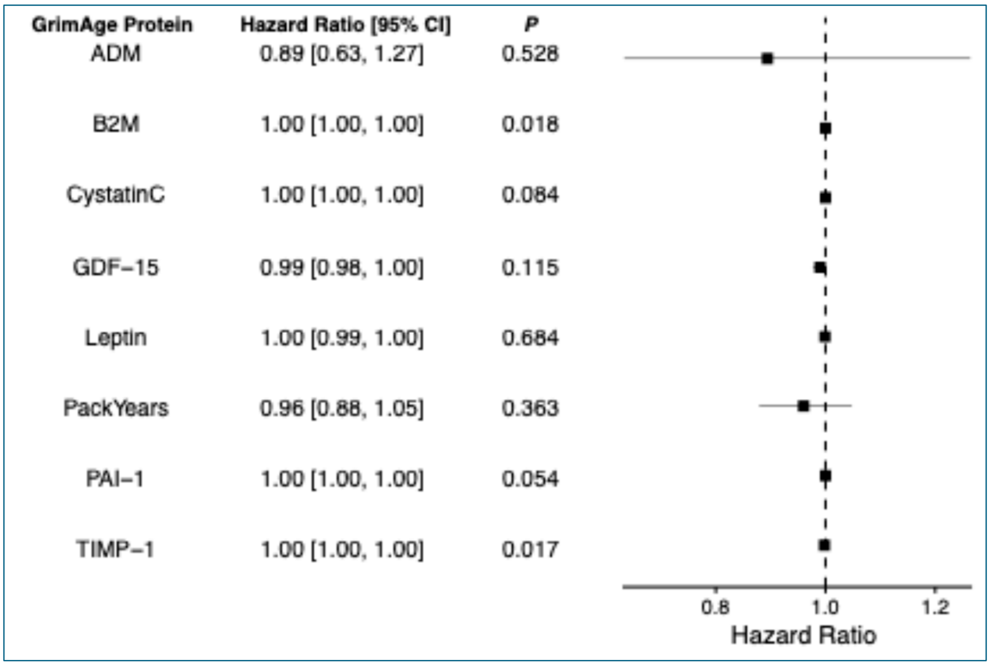

B.

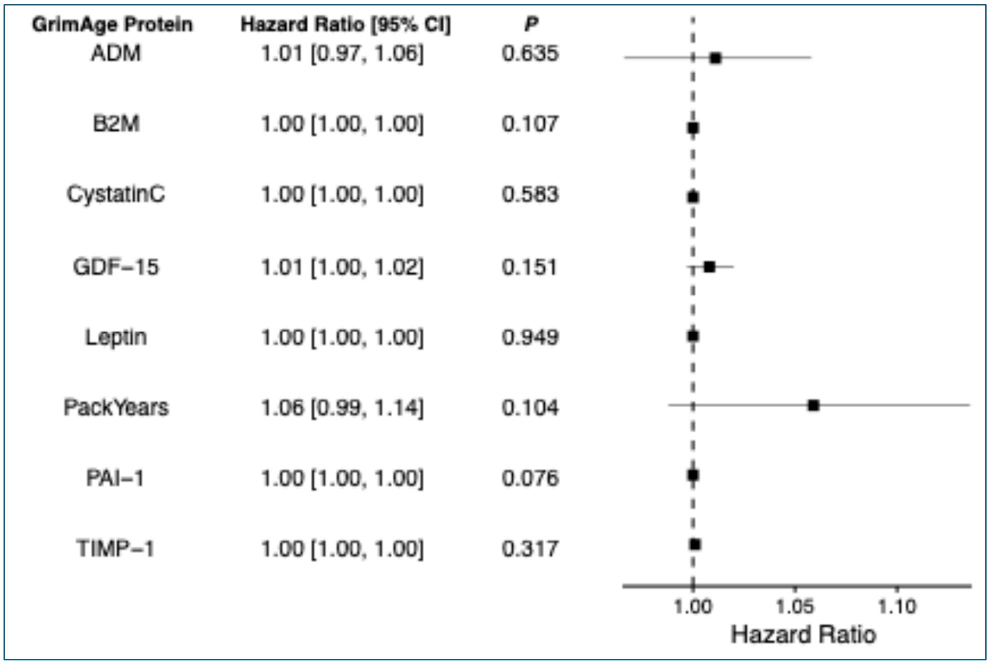

ADM, adrenomedullin; B2M, beta-2 microglobulin; GDF-15, growth differentiation factor 15; PAI-1, plasminogen activation inhibitor-1; TIMP-1, tissue inhibitor metalloproteinase 1

Supplemental Figure S8. Hazard ratios for the associations between the blood-based plasma proteins of GrimAge and overall survival in R/M HNSCC among patients (**A**) less than 65 years of age and (**B**) 65 years of age or older

A.

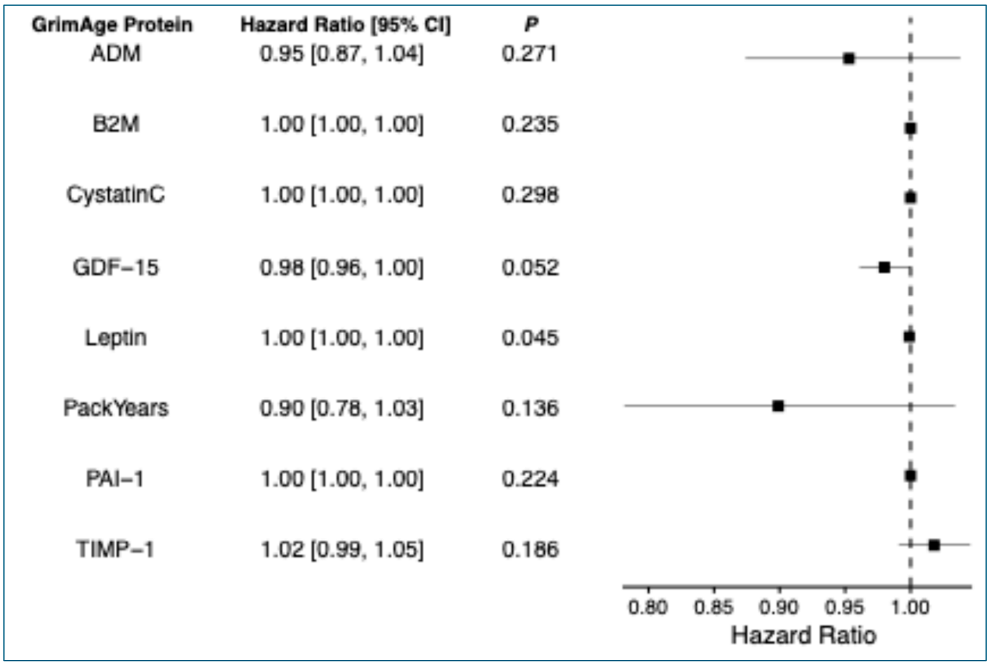

B.

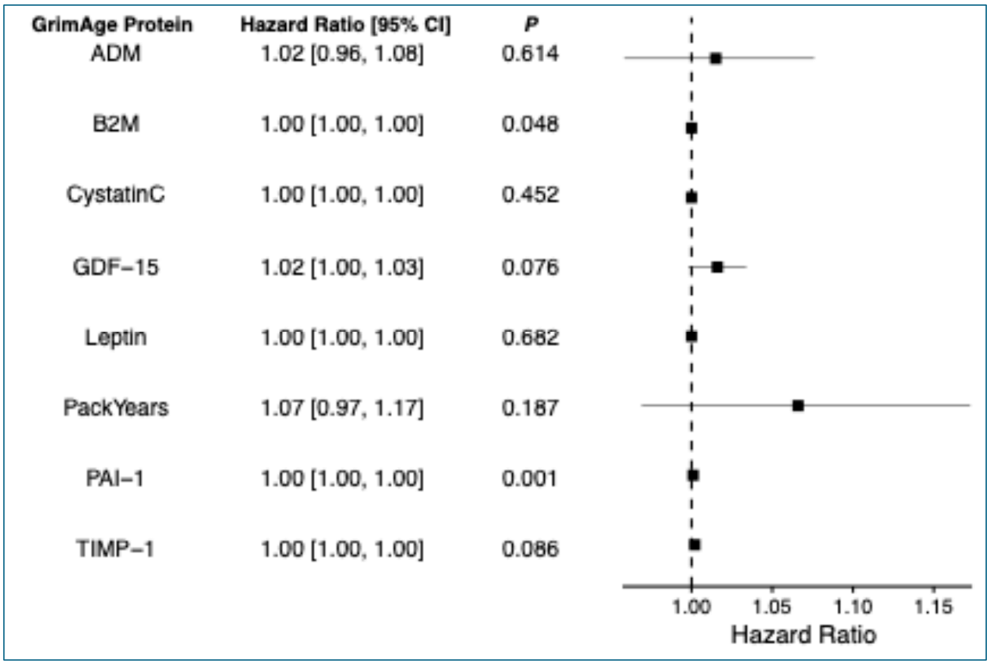

ADM, adrenomedullin; B2M, beta-2 microglobulin; GDF-15, growth differentiation factor 15; PAI-1, plasminogen activation inhibitor-1; TIMP-1, tissue inhibitor metalloproteinase 1
